# Stem-Like Signature Predicting Disease Progression in Early Stage Bladder Cancer. The Role of E2F3 and SOX4

**DOI:** 10.3390/biomedicines6030085

**Published:** 2018-08-02

**Authors:** Joaquim Bellmunt

**Affiliations:** 1 Department of Medical Oncology, Hospital del Mar, IMIM (PSMAR-Hospital del Mar Research Institute), 08003 Barcelona, Spain; jbellmunt@imim.cat; Tel.: +34-933-160-750; 2 Harvard Medical School, Boston, MA 02115, USA

**Keywords:** bladder cancer, bladder cancer stem cells, SOX-4, E2F3, non-muscle invasive bladder cancer, EMT

## Abstract

The rapid development of the cancer stem cells (CSC) field, together with powerful genome-wide screening techniques, have provided the basis for the development of future alternative and reliable therapies aimed at targeting tumor-initiating cell populations. Urothelial bladder cancer stem cells (BCSCs) that were identified for the first time in 2009 are heterogenous and originate from multiple cell types; including urothelial stem cells and differentiated cell types—basal, intermediate stratum and umbrella cells Some studies hypothesize that BCSCs do not necessarily arise from normal stem cells but might derive from differentiated progenies following mutational insults and acquisition of tumorigenic properties. Conversely, there is data that normal bladder tissues can generate CSCs through mutations. Prognostic risk stratification by identification of predictive markers is of major importance in the management of urothelial cell carcinoma (UCC) patients. Several stem cell markers have been linked to recurrence or progression. The CD44v8-10 to standard CD44-ratio (total ratio of all CD44 alternative splicing isoforms) in urothelial cancer has been shown to be closely associated with tumor progression and aggressiveness. ALDH1, has also been reported to be associated with BCSCs and a worse prognosis in a large number of studies. UCC include low-grade and high-grade non-muscle invasive bladder cancer (NMIBC) and high-grade muscle invasive bladder cancer (MIBC). Important genetic defects characterize the distinct pathways in each one of the stages and probably grades. As an example, amplification of chromosome 6p22 is one of the most frequent changes seen in MIBC and might act as an early event in tumor progression. Interestingly, among NMIBC there is a much higher rate of amplification in high-grade NMIBC compared to low grade NMIBC. *CDKAL1*, *E2F3* and *SOX4* are highly expressed in patients with the chromosomal 6p22 amplification aside from other six well known genes (*ID4*, *MBOAT1*, *LINC00340*, *PRL*, and *HDGFL1*). Based on that, *SOX4*, *E2F3* or 6q22.3 amplifications might represent potential targets in this tumor type. Focusing more in *SOX4*, it seems to exert its critical regulatory functions upstream of the Snail, Zeb, and Twist family of transcriptional inducers of EMT (epithelial–mesenchymal transition), but without directly affecting their expression as seen in several cell lines of the Cancer Cell Line Encyclopedia (CCLE) project. *SOX4* gene expression correlates with advanced cancer stages and poor survival rate in bladder cancer, supporting a potential role as a regulator of the bladder CSC properties. *SOX4* might serve as a biomarker of the aggressive phenotype, also underlying progression from NMIBC to MIBC. The amplicon in chromosome 6 contains *SOX4* and *E2F3* and is frequently found amplified in bladder cancer. These genes/amplicons might be a potential target for therapy. As an existing hypothesis is that chromatin deregulation through enhancers or super-enhancers might be the underlying mechanism responsible of this deregulation, a potential way to target these transcription factors could be through epigenetic modifiers.

## 1. Urothelial Stem Cells

Normal adult stem cells in the basal layer of the urothelium can regenerate and proliferate to restore urothelial integrity after damage [1]. These basal urothelial stem cells have a high nuclear to cytoplasmic ratio, express CD44, laminin receptor (LR), β1 and β4 integrins, and specific “basal” cytokeratins (CK-5/14, CK-17) [2]. Phenotypically similar populations of cells have been isolated from urothelial cancer cell lines and primary tumors [3].

The tissue-specific stem cells of the normal urothelium have been proposed to reside in the basal layer, responsible for the maintenance of tissue homeostasis and renewal. The basement membrane serves as a nidus for epithelial stromal interactions that are essential for stem cell preservation [4]. Alternatively, it has also been suggested that at least two independent pools of urothelial stem cells exist [1].

In the attempt to identify and target tumor-initiating cell populations, the similarities between normal and tumor stem cells of the same tissue have been employed. Many molecules expressed by normal stem cells have been found in their malignant counterparts [1]. For example, the embryonic stem cell marker OCT3/4, akey regulator of self-renewal, showed high expression in human bladder cancer and the level of expression correlated with tumor aggressiveness and progression rate in patients [5,6].

Another marker, CD44 is one of the most prominent stem cell markers. CD44+ cells are located in the basal layer of the normal urothelium as well as in the UCC [7]. CD44 is a cell surface molecule that has been related to multiple functions including cell differentiation, cell proliferation, cell migration and angiogenesis. Other potential functions include presenting cytokines, chemokines, and growth factors to the corresponding receptors, docking of proteases at the cell membrane and cell survival signaling.

## 2. Cancer Stem Cells (CSC) in Bladder Cancer

The understanding of the role of stem cells in tissue biology is the base of the proposal that cancers might similarly develop from a progenitor pool (the “cancer stem cell (CSC) hypothesis”). This idea supports that CSCs differentiate into cancer cells and therefore to tumors, like normal adult tissues would arise from a specific stem-like cell population. These would drive tumor growth and ability to metastasize, as well as resistance to conventional antitumor therapy.

Cancer stem cells (CSC) were defined in 2006 as malignant cells with an ability to renew and differentiate to form all of the cell types in a given tumor (American Association for Cancer Research Workshop 2006 [8]. In 2009 bladder cancer stem cells (BCSCs) were identified for the first time via the markers used to isolate normal stem cells [9], and their existence was supported by subsequent studies [10]. Urothelial cancer stem cell comprises a tumor cell subpopulation with tumor-initiating potential, self-renewal, clonogenic and proliferative capacity. These cells are observed in normal urothelium in tumors and have the ability to conserve cellular heterogeneity via differentiation and hierarchical tissue organization.

The rapid development of the CSC field, together with powerful genome-wide screening techniques, have provided the basis for the development of future alternative and reliable therapies.

One of the characteristics of CSC is the stemness; i.e. the ability to self-renew and differentiate [11], for which several signaling pathways are overactivated such as *JAK/STAT*, *Wnt/β-catenin*, *Nanog*, and *Notch*, depending on the tumor type [12,13]. CSCs sometimes phenotypically resemble non-stem cancer cells and this has been related to therapy resistance. Non-stem cancer cells can acquire stemness by dedifferentiation in response to multiple stimuli, possibly including conventional cancer therapies [14,15].

Some studies hypothesize that bladder CSCs (BCSCs) do not necessarily arise from normal stem cells, but might derive from differentiated progenies following mutational insults and acquisition of tumorigenic properties [1]. Recent studies support that normal bladder tissues might transform into CSCs through mutations in urothelial stem cells, basal cells, intermediate cells and terminally differentiated umbrella cells [10]. Old studies were supporting that CSCs may arise from normal stem cells having suffered gene mutations instead of deriving from differentiated progenies [16]. Although this concept was abandoned, it has now emerged again when recent papers have shown that, co-mutagenesis of ARID1A, GPRC5A and MLL2 by CRISPR/Cas9 technology significantly enhanced self-renewal and tumor initiation properties of BCSCs [17].

### CSC Markers in Bladder Cancer

BCSCs are heterogenous and identification of these cells is crucial since they are integral to the initiation, high recurrence and chemoresistance of bladder cancer.

The commonly reported cancer stem cell markers and pathways that have been evaluated include CD44, CD133, ALDH1, SOX2, SOX4, BMI1, EZH1, PD-L1, MAGE-A3, COX2/PGE2/STAT3, AR, and autophagy. Other cell surface markers to identify and isolate BCSCs, include CD67LR, EMA, and BCMab1 [18].

Epithelial-mesenchymal transition-related pathways (Shh, Wnt/β-catenin, Notch, PI3K/Akt, TGF-β, miRNA) are also involved. Their regulative role in the development and differentiation of BCSCs has been confirmed in vitro by functional analyses (e.g., cell migration, colony formation, sphere formation), and in vivo xenograft experiments [19]. In addition to these well know signaling pathways involved in BCSC (hedgehog, Notch and Wnt) aberrant activation of the STAT3 and JAK-STAT signaling pathway have been described [20,21,22,23,24,25].

Based on all these data, the use of a combination of markers could help refine the CSC phenotype in BC and in other tumors.

## 3. CSC as Markers of Progression

In clinical practice we are still missing reliable prognostic markers to identify those papillary tumors more prone to progress to being muscle-invasive. In this context, the use of CSC markers as a prognostic tool has been limited by the functional and phenotypic heterogeneity of CSCs populations.

Along the same line, recurrence of NMIBC has been related to the presence of undifferentiated cells exhibiting stem-like properties, the so-called cancer stem cells (CSCs) [26,27]. These tumor initiating undifferentiated cells can undergo unlimited self-renewal and have been shown to reform the tumors when implanted in immunocompromised mice [28,29].

Several stem cell markers have been found to be linked to recurrence or progression. One example is the CD44v8-10-to-standard CD44-ratio (total ratio of all CD44 alternative splicing isoforms) in urothelial cancer that has been shown to be closely associated with tumor progression and aggressiveness [4,30]. ALDH1, has also been reported to be associated with BCSCs and a worse prognosis in a large number of studies [31,32].

## 4. Stem Cell Differences between NMIBC and MIBC

Most bladder caners are non-invasive urothelial papillary tumors. These display high recurrence rates after resection but only rarely infiltrate the bladder wall or develop metastasis. However, 10–30% of them develop into high-grade invasive tumors and it is critical to identify genetic defects and predictive markers for risk stratification. For example, NMIBC which account for 70–80% of human UCC cases are frequently associated with activating mutations of some proto-oncogenes, of which fibroblast growth factor receptor 3 (FGFR3) and HRAS are most prevalent. These mutations are present in up to 75% and 30% of the papillary tumors, respectively, and are linked to different outcome.

In contrast, the remaining 20–30% of human UCC are constituted by MIBC, related to loss of p53, RB, PTEN and activation of EMT-TFs (Epithelial Mesenchymal Transition-Transcription Factors). In this subset, proliferation seems to be mediated by E2F3 (controlled by miR-125b) and EMT that is regulated by the miR-200 family [1,33]. All these mutations result in genomic instability and an anti-apoptotic phenotype, which enables tumor progression through accumulation of mutations.

Both the high recurrence rates and tumor heterogeneity of bladder cancer have been related to bladder cancer stem cells [34]. Attempts to isolate BCSCs based on the basal cell surface marker CD44 expression-despite being closely associated with tumor progression and aggressiveness- showed substantial variation among basal tumor subtypes and have been at least unsuccessful in non-muscle-invasive tumors [35,36,37].

Trying to link grade and stage with stem-like properties, Brandt et al. proposed that BCSCs, for low-grade papillary/noninvasive and high-grade flat/invasive UCC, have a different origin associated with a distinct genetic background. His gene-profiling study showed that noninvasive urothelial carcinomas predominantly express mRNA-encoding markers of differentiated urothelial cells; among others, the superficial/umbrella cell marker uroplakin 2 and the cell adhesion proteins LAMB3 and ITGB4 [34].

Teixeira et al. [38] showed that distinct cell subsets of muscle-invasive BC can show molecular features of stem-like cells with an aggressive phenotype, enhanced chemoresistance and tumor-initiating ability. In their report [38], they used distinct stem cell-related markers, such as embryonic transcription factors (OCT4 (POU5F1), SOX2 and NANOG), ABC transporters (PGP (ABCB1) and BCRP (ABCG2)), aldehyde dehydrogenase isoforms (ALDH1A1, ALDH2 and ALDH7A1), and basal urothelial stem cell markers (CD44, CD47 and KRT14). The authors showed a significant co-upregulation of CD44 and of basal-type KRT14. The KRT14 cytokeratin is a primitive stem cell marker precursor to KRT5 and KRT20 in urothelial differentiation and has been associated with tumor recurrence and poor overall survival, independently of known clinical and pathological variables [39,40]. They analyzed gene expression patterns in primary clinical samples and could identify a two-gene stem-like signature (SOX2 and ALDH2) potentially useful to identify muscle-invasive tumors that are more susceptible to progression or metastasis. SOX4 or E2F3 were not analyzed in this study.

Based on this study [38], the role of CSCs as driving forces in the pathogenesis and relapse of invasive BC is reinforced based on expression of at least two stemness-related markers in muscle-invasive tumors. This supports the interest of identifying, novel therapeutic approaches considering CSCs as a target population. This signature could help to prospectively identify BC patients that could benefit from a more aggressive therapeutic intervention targeting CSCs at earlier time points. Prospective confirmation of these findings will be required.

## 5. Role of Amplification of 6p22 in Bladder Cancer

Even though knowledge about copy number alterations in BC is limited, amplification of chromosome 6p22 is frequently described [41,42]. As was shown by Shen et al. [31] with The Cancer Genome Atlas (TCGA) dataset and cBio Cancer Genomics Portal analysis, they observed amplification of chromosome 6p22 in 18% of bladder cancer patients. Some authors have described that amplification of chromosome 6p22 was significantly associated with MIBC in contrast to NMIC [43,44,45]. This observation was subsequently supported by a subsequent paper, by Shen et al. observing that the amplification of chromosome 6p22.3 was associated with the 22% of MIBC in contrast to 9% of NMIBC (*p* = 0.04) [45]. Interestingly, they also observed a much higher rate of 6p22.3 amplification in high-grade NMIBC (13%; 12/93) compared to low-grade NMIBC (2%; 1/47). Tumor depth of invasion in MIBC was also associated with 6p22.3 amplification (*p* = 0.12). However, they failed to show a significant association of amplification (35/181; 19.2%) with survival (log-rank *p* = 0.438) for the 181 MIBC patients who underwent a cystectomy for curative intent. The authors hypothesize that 6p22.3 amplification might act as an early event in tumor progression. This report supports that amplification 6p22.3 together with the standard pathological factors—such as grade, depth of invasion (pT), and positive nodes (pN)—is associated with a more aggressive phenotype [45]. When examining the 6p22.3 region of amplification eight known genes (ID4, MBOAT1, E2F3, CDKAL1, SOX4, LINC00340, PRL, and HDGFL1) are present [31]. RNA-seq results showed that CDKAL1, E2F3 and SOX4 in the 6p22.3 region were highly expressed in patients with the chromosomal 6p22 amplification. E2F3 has been characterized as a potential cell proliferation effector of 6p22 amplification. Knockdown of E2F3 was observed to inhibit cell proliferation in a 6p22.3-dependent manner while knockdown of CDKAL1 and SOX4 did not affect cell proliferation [45].

“Oncogene addiction”, a term first coined in 2000 by Bernard Weinstein, reveals a possible “Achilles’ heel” within the cancer cell that can be exploited therapeutically. One could hypothesize that 6p22.3 could be explored as a potential “Achilles’ heel” and this region of amplification as an area of “amplicon addiction” that could be modulated epigenetically.

### Role of 6p22 Amplification in Cell Lines

Three MIBC cell lines (5637, TCC-SUP and HT1376) that contain amplification of the 6p22 region have been described [45]. E2F3a, E2F3b, CDKAL1 and SOX4 are highly expressed in the 6p22-amplified 5637 cells. In TCC-SUP and HT-1376 cells the E2F3a and E2F3b mRNA levels were similar to those in the control of non-6p22-amplified cells and amplification of 6p22 did not correlate with gene expression values. SW780 and J82 cells showed high expression of CDKAL1 and RT-112, and RT-112-D21 cells showed high expression of SOX4. However, none contained the amplified 6p22 region.

Proliferation of the 5637-bladder cancer cell line was found to be highly dependent on the 6p22.3 amplicon, particularly of the E2F3 gene. In this cell line, cell proliferation was reduced when E2F3a or E2F3b were knocked down compared to no effect with knockdown of SOX4 and CDKAL1 [43,44,46]. CCND1 was also down regulated in response to shE2F3a.

The authors confirmed that cell proliferation induced by E2F3 is dependent on chromosomal 6p22 amplification repeating the experiment in 253J and T24, two other cell lines without 6p22 amplification in which knockdown of E2F3 did not inhibit cell proliferation. This supports that the E2F3 role depends on the presence of chromosomal 6p22 amplification, maybe through an “amplicon addiction” mechanism [45].

## 6. Role of E2F3 in Bladder Cancer

In human bladder cancer, amplification of the E2F3 gene, located at 6p22, is associated with overexpression of its mRNA and high expression of the E2F3 protein. This overexpression is seen in over one third of primary transitional cell carcinomas and increases with tumor stage and grade.

Because of the role E2F3 in cell cycle progression, these findings support that the E2F3 gene represents a candidate bladder cancer oncogene activated by DNA amplification and overexpression [43,44,47].

Additionally, CCND1—a key cell cycle regulator specifically for the G1-to-S phase—has been identified as a potential target of E2F3 [48]. Binding of cyclin D1 to cyclin-dependent kinase (CDKs) leads to retinoblastoma protein (pRb) phosphorylation followed by release of E2F transcription factors allowing G1- to S-phase progression. Inhibition of CCND1 and CDK4/6 may potentially reverse the oncogenic effect elicited by E2F3 amplification in TCC-UB cell lines with amplified 6p22. (Palbocyclib testing is ongoing).

Besides, increased tumor recurrence and progression in patients with NMIBC has been associated with increased E2F and Ezh2 expression in a study that provides a genetically defined model for human high-grade NMIBC. The report shows that the Rb–E2F–Ezh2 axis can promote tumor development when disrupted [49].

## 7. SOX4

SOX4 is a member of the SOX (SRY-related HMG-box) family of transcription factors involved in organogenesis of the heart, pancreas, and brain, and in T lymphocyte differentiation. SOX4 gene expression is upregulated in many cancer types, and increased SOX4 activity contributes to cellular transformation, cell survival, and metastasis [50].

SOX family of proteins are found in all metazoans, and high expression of SOX2 has been used to recognize the presence of CSCs. This approach has been applied in some studies of urothelial carcinoma [21,32,51,52].

## 8. SOX4 in Tumors

High levels of *SOX4* gene expression have been reported in diverse human cancers including leukemia, colorectal cancer, lung cancer and breast cancer. It has been related to both apoptosis (leading to cell death) and to tumorigenesis suggesting a role in the development of these malignancies [53,54] through the epithelial-to-mesenchymal transition (EMT) mechanism [53].

A systematic review and meta-analysis of *SOX4* as a potential prognostic factor in human cancers was carried out analyzing the expression status of *SOX4* in twenty kinds of human cancers at a protein level (The Human Protein Atlas). The positive rate of *SOX4* expression was about 78% in overall cancer tissues. The meta-analysis showed that SOX4 overexpression correlated with a poor overall survival with a pooled hazard ratio (HR) of 1.67 (95% CI 1.01–2.78). The study concluded that *SOX4* is a potential prognostic biomarker in human cancers [55].

## 9. SOX4 in Bladder Cancer

*SOX4* gene expression was increased 2.2-times in bladder tumors compared to normal tissue by immunohistochemistry and real-time PCR [56]. Immunostaining, used to confirm the presence of protein, showed significant differences between bladder tumors and normal bladder tissue (*p* = 0.001). Altogether these data suggest that *SOX4* gene may have a role in bladder cancer tumorigenesis [56].

Based on gene expression profiling, another report showed correlation of high *SOX4* expression with advanced cancer stages and poor survival, supporting again a potential role of *SOX4* as a regulator of the BCSC properties that may serve as a biomarker of the aggressive phenotype in bladder cancer [31].

One study has provided contradictory result, [57] potentially related to differences in the SOX4-specific antibody used. This study conducted in 2360 clinically annotated bladder tumors using tissue microarray found unexpectedly, a correlation (*p* < 0.05) between strong *SOX4* expression and increased patient survival.

## 10. SOX4 and EMT

BCSCs are enriched with elevated levels of genes acting in EMT [58]. EMTs underlie the pathophysiology by which sessile, epithelial cells lose polarity and cell–cell adhesion, and transition to motile, mesenchymal stem cells. This process increases migratory and invasive potential during organismal development and is key for progression of epithelial tumors to metastatic cancers.

Activation of EMT program in cancer cells switches the CSCs with stationary phenotype to migratory phenotype. Cancer cells can then enter the blood circulation, extravasate, and eventually metastasize to target organs [1].

EMT may render cancer cells with cancer stem cell properties, and/or stimulate the expansion of malignant BCSC population, giving rise to a more aggressive tumor type. It is therefore critical to explore for the connection between EMT and cancer stemness to assess their potential implications in bladder cancer therapy [1].

Breast cancer studies have suggested that *SOX4* induces epithelial-to-mesenchymal transition (EMT) and cooperates with the RAS oncogene in cancer progression [59]. In breast cancer, *SOX4* is believed to play a critical role in the early stages of the malignant progression of breast cancer. The same might happen in other tumor types like bladder cancer [50].

Tewari went on to identify *SOX4* target genes validated by qRT-PCR in response to siSOX4, including *CCND1*, *CDK1*, *FGFR1*, *FGFR3*, *MYB* and *MYC*. The authors also found that tight junction proteins, like CRB3, TJP1, TJP3, were specifically up-regulated in response to knock down of *SOX4*. This gene expression profiling data indicates that SOX4 is involved in other signaling pathways and cellular processes that regulate cell migration and invasion besides it’s known role in classic cell cycle regulation. Interestingly, their findings support that SOX4 exerts its regulatory functions upstream of the Snail, Zeb, and Twist family transcriptional inducers of EMT, but without directly affecting their expression as seen in several cell lines of the CCLE project. [50] They concluded that SOX4 is a master regulator of epithelial-mesenchymal transition by governing the expression of the epigenetic modifier, Ezh2 [50].

Shen et al. [31] demonstrated that, knockdown of *SOX4* reduced sphere formation, aldehyde-dehydrogenase-high (ALDHhigh) cell population and potential of tumor formation.

As of today, the detailed mechanisms of SOX in the regulation of BCSCs are far from clear to us. To further update the SOX4 dependencies in different bladder cancer cell lines Table 1, describe recent dependency data based on CERES score and CRISPR Avana.

## 11. Epigenetics and CSC

Beyond common CDs like CD44 and CD133, ALDH1, and SOX, EMT-related markers like polycomb repression complex (PRC) related pathway have its role on CSC biology [60]. PRC2 mediates the trimethylation of histone H3 at lysine 27, a hallmark of gene silencing and facultative heterochromatin formation, and its dysregulation is linked to human diseases. PRC2 consists of four core subunits, *EZH2*, *Eed*, *SUZ12* and Rbbp4. Among these, EZH2 is the catalytic subunit that requires Eed and SUZ12 for catalysis [61]. EZH2 plays a role in tumor invasiveness, colony formation and migration and are related to the expression of CSC-related genes (CD44, KLF4, OCT4 and ABCG2). *EZH2* was found to be regulated by *E2F1* [62], which was previously linked to aggressiveness and prognosis of bladder cancer [62,63].

Bmi-1 (a member of PRC1) serves as the gene silencer that induces cellular senescence and cell death, and it can contribute to cancer when improperly expressed. Bmi-1 overexpression (mostly due to gene amplification) leads to *INK4A*/*ARF* locus repression and consequent inactivation of Rb and p53 [64]. In contrast to many studies on the potential involvement of Bmi-1 in the oncogenesis of various lymphomas and leukemias, there is still a lack of knowledge about its role in the pathogenesis of many solid tumors, including UCC. A recent study has confirmed overexpression of Bmi-1 protein in BC correlated with tumor classification, recurrence, TNM stage, and survival, proposing it as a possible prognostic marker. Bmi-1 protein was up regulated to a much greater extent than Bmi-1 mRNA in cancer tissue, suggesting deregulation at the post-transcriptional level [65] despite that some authors reported no significant Bmi-1 mRNA expression [66].

A genomics approach revealed an 11-gene signature (including BMI1) that consistently displayed a stem-cell-resembling expression profile in distant metastatic lesions of different cancers, including bladder cancers [67].

Other data suggest that Bmi-1 overexpression is probably not a primary event in the genetics of BC, but is involved in the progression of the tumor [68].

Other members of the Polycomb family genes have also been shown to correlate with disease development, presenting novel potential targets for therapy. For example, CBX7 that inversely correlated with the tumor stage and grade progression in BC, or *EZH2* expression that showed a significant increase in UCC specimens and bladder cancer cell lines [69,70]. Interestingly, the Polycomb group of proteins are commonly abnormally overexpressed years prior to cancer pathology, making early targeted therapy an option to reverse tumor formation [4,71].

BCSCs function also interacts with epigenetics. Histone modification and chromatin rearrangement mutations are frequently present in CSCs [72], and promote tumor initiation [73] influencing genome integrity [74] or leading to epigenetic reprogramming [75].

Chromatin modifying genes are frequently mutated in bladder cancer. This chromatin dysregulation might be responsible of the epigenetic modification through enhancers or super-enhancers in different areas of the genome. The amplicon in chromosome 6 that contains SOX-4 and E2F3 is frequently found amplified in bladder cancer might be epigenetically regulated and might be a potential target for therapy.

## 12. Epigenetics and SOX4

Genetic and epigenetic alterations have been linked to transitional cell carcinoma of the urinary bladder (TCC). The transcription factor *SOX4* has been identified as a master regulator of EMT. It controls a number of EMT-relevant genes in addition to EZH2, a critical *SOX4* target gene during EMT. There seems to be interplay between transcriptional and epigenetic control during EMT which suggest that the inhibition of EZH2 could be an attractive avenue for the therapeutic intervention of progression [50].

The histone methyltransferase Ezh2 (Enhancer of Zeste homolog 2) is a component of the Polycomb (PcG) repressive complex 2 (PRC2) which epigenetically regulates genes involved in cell fate decisions. Ezh2 specifically trimethylates nucleosomal histone H3 at lysine 27 (H3K27me3) an epigenetic modification associated with gene silencing [76].

Of note, in early-stage lymph-node-negative breast cancer, the concomitant high expression of Ezh2 and *SOX4* significantly correlated with poor metastasis-free survival [50].

There seems to be a complex gene regulatory network driving the increased expression of *SOX4* during the early phases of TGFb-induced EMT. These findings are indeed hypothesis-generating, and warrant further investigation to unravel the action of transcription factors and epigenetic regulators in driving the transcriptional reprogramming underlying progression to subsequent stages.

## 13. Therapeutic Implications. Need to Eradicate Csc in Therapy

CSCs are attributed to have tumorigenic potential and ability to dictate invasion and metastatic progression as well as enhanced resistance to therapy. Among other mechanisms for these actions are the quiescence or slow cycle kinetics, enhanced DNA repair mechanisms and overexpression of multidrug resistance-type membrane transporters. All these processes can contribute to the failure of existing therapies [77,78]. Considerable evidence associates CSCs to high recurrence rates and poor survival and failure of adjuvant treatment in patients with MIBC [10,38,79].

Although metastatic bladder cancer is highly responsive to chemotherapy with cisplatin, only a small cohort of patients (10–20%) respond completely as evident by eradication of BCSCs in metastasis and prolonged survival of patients [1].

These remaining cisplatin-resistant bladder cancer cells include the existence of CSC-like cells that display higher levels of Bmi1 and Nanog expression, EMT characteristics, CSC marker expression, and sphere-forming capacity, conferring them a role in progression and drug resistance of bladder cancer [80].

Inability of currently widely used anti-cancer therapies to kill CSCs is one of the probable reasons for their failure and tumor relapse. Underlying CSCs remain viable as quiescent BCSC in patients with metastatic disease. This tumor dormancy is a key limiting factor in the treatment of metastatic diseases and understanding the mechanisms behind stem cell proliferation and differentiation could lead to the development of new anti-cancer strategies. These, alone or in combination could successfully target the BCSC population by inhibiting the maintenance of stem cell state as well as by killing the bulk tumor cell population [1].

Few targeted therapies have shown promising results in bladder cancer [81,82]. In contrast, immunotherapy (e.g., immune checkpoint blockade) has provided good objective responses and prolonged survival [83,84,85]. Experimental monoclonal antibodies against some surface markers (such as 67LR and CD47) have already given some promising results in human xenografts and in vitro studies. As an example, CD47 is highly expressed on UCC and functions as a ligand of the SIRP inhibitory molecule expressed on phagocytes. Blockade of CD47 by a monoclonal antibody resulted in efficient and specific macrophage engulfment of bladder cancer cells in vitro [4].

Inhibition of Ezh2 function could also be an interesting alternative for therapeutic intervention during tumor progression as shown by promising early results with the Ezh2 inhibitor 3-deazaneplanocin (DZNep) [86,87]. Results from clinical trials are awaited [50].

Another potential agent, honokiol, a biologically active biphenolic compound isolated from *Magnolia officinalis*, has been shown to inhibit cancer cell proliferation, survival, cancer stemness, migration and invasion, through the downregulation of EZH2 expression levels, along with reductions in the expression of matrix metalloproteinase 9, CD44, SOX2 and the induction of tumor suppressor miR-143 in bladder cancer [52].

Similarly CD274, another CSC marker of relevance, showed useful results in a study focusing on CD274 (PD-L1) in cholangiocarcinoma through in vivo and in vitro experiments [88]. The results of the study provided direct evidence of the participation of PD-L1 in the activity of CSCs. More research into the mechanisms through which PD-L1 affects BCSCs is required to illustrate how CSCs participate in these checkpoint immunomodulatory actions [19]. Being PD-L1 a CSC marker, can help to explain the long lasting responses seen with immunotherapy.

## 14. Conclusions

There is now evidence that E2F3 in 6p22-amplified bladder cancer is a potential oncogene of critical importance. Also, the 6p22.3 amplicon itself might be a potential target for therapeutic intervention.

Future studies are needed to determine the interactions of E2F3 with other genes in the 6p22 amplicon such as *SOX4*. In addition, other potential oncogenes, such as CCND1 not in the 6p22 amplicon but yet frequently altered in bladder cancer and dependent on E2F3 could be secondarily impacted.

Importantly, the critical biological role of *SOX4* in EMT has raised the question of whether *SOX4* contributes to malignant tumor progression and metastasis. The fact that the 6p22.3 amplicon is being frequently amplified in MIBC and contains the *SOX4* transcription factor creates the speculation that *SOX4* might be the underlying force of progression from high grade NMIBC to MIBC.

## Figures and Tables

**Table 1:** *SOX4* dependencies, based on CERES score—CRISPR Avana: *SOX4* overexpression and high CN: 5637, TCCSUP; *SOX4* overexpression, NO amplification: RT4; *SOX4* amplification, NO overexpression: HT1376.

Cell Lines Dependencies SOX4	SOX4 Dependency Score (CERES) (CRISPR (Avana))	SOX4 Copy Number (log2 Relative to Ploidy) (Copy Number)	SOX4 RPKM (log2) (Expression)	SOX4 Mutations
HT1376_URINARY_TRACT	−0.588396496	2.151	−0.035608762	
5637_URINARY_TRACT	−0.530627172	3.0085	6.597139609	
TCCSUP_URINARY_TRACT	−0.528520427	2.626	6.485082053	
RT4_URINARY_TRACT	−0.514677372	0.0892	5.03949332	
JMSU1_URINARY_TRACT	−0.308209725	2.0774	3.753360644	
HT1197_URINARY_TRACT	−0.210962969	0.3168	4.563195967	
KMBC2_URINARY_TRACT	−0.178771111	0.3268	6.645048373	
VMCUB1_URINARY_TRACT	−0.165715336	−0.1723	1.701167541	
BFTC905_URINARY_TRACT	−0.159836964	0.203	4.275478678	
KU1919_URINARY_TRACT	−0.128052003	−0.0704	1.944734781	
RT11284_URINARY_TRACT	−0.122120917	0.0121	5.587819212	
UMUC1_URINARY_TRACT	−0.108931406	−0.4586	5.197921205	In_Frame_Ins (p.168_168G>GG)
SCABER_URINARY_TRACT	−0.078074986	−0.0499	1.724986352	
639V_URINARY_TRACT	−0.05968828	0.1646	2.397989104	
647V_URINARY_TRACT	−0.030569379	0.682	5.697895003	
UMUC3_URINARY_TRACT	−0.027795422	0.1803	1.450005009	
BC3C_URINARY_TRACT	−0.001039065	−0.0051	2.279004684	
CAL29_URINARY_TRACT	0.036924246	0.0177	5.603229902	
RT112_URINARY_TRACT	0.085456564	0.0319	4.993583341	
T24_URINARY_TRACT	0.105449672	−0.2206	2.681145626	
253J_URINARY_TRACT	-	-	4.892009171	
